# Primary pituitary stalk mucosa-associated lymphoid tissue lymphoma: a case report and literature review

**DOI:** 10.3389/fneur.2023.1193391

**Published:** 2023-07-06

**Authors:** Shihao Cai, Juexian Xiao, Peng Chen, Haitao Luo, Zujue Cheng

**Affiliations:** ^1^Department of Neurosurgery, The Second Affiliated Hospital of Nanchang University, Nanchang, Jiangxi Province, China; ^2^Institute of Neuroscience, Nanchang University, Nanchang, Jiangxi Province, China

**Keywords:** primary CNS lymphoma, MALT lymphoma, pituitary stalk mass, surgery, hormone replacement therapy

## Abstract

**Background:**

Primary extranodal mucosa-associated lymphoid tissue (MALT) lymphoma in the sellar region is a rare indolent B-cell lymphoma.

**Case presentation:**

A newly diagnosed patient with MALT lymphoma originating from the pituitary stalk is reported. A space-occupying lesion in the sellar region was found in a 24 year-old man who had no clinical symptoms except for those relating to a sex hormone disorder (rising estrogen and falling androgen) identified during a pre-employment physical examination. MALT lymphoma was diagnosed pathologically. Radiotherapy and chemotherapy were proposed after surgery. However, the patient selected androgen replacement therapy only rather than chemoradiotherapy. Over the next 3 months, no visual disturbance, headache, cranial nerve abnormality, or other symptoms occurred.

**Conclusion:**

Primary sellar region MALT lymphoma is an extremely rare disease. The differential diagnosis of sellar and parasellar masses should include primary sellar region MALT lymphoma. Early detection and treatment of this lymphoma can effectively improve the prognosis.

## 1. Introduction

Marginal zone lymphoma (MZL) is a low-grade, non-Hodgkin lymphoma. The World Health Organization divides it into three types: extranodal marginal zone B cell lymphoma (EMZL), intranodal lymphoma, and intrasplenic lymphoma (1). EMZL is an indolent lymphoma that can appear at any extranodal location. Isaacson and Wright initially defined EMZL as a low-grade lymphoma of the gastrointestinal tract (2). However, according to the location, EMZL can be divided into gastric and non-gastric mucosa-associated lymphoid tissue (MALT) lymphoma. The latter frequently appears in ocular appendages, skin, thyroid, lungs, salivary glands, and breasts (3) but is extremely rare in the sellar region; most central nervous system (CNS) cases occur in the dura or the brain parenchyma (4–8). A case of MALT lymphoma originating from the pituitary stalk is reported here, and the literature was searched for case studies to review how to diagnose and treat MALT lymphoma in the sellar region.

## 2. Case presentation

### 2.1. Medical history

Sparse body hair and pale skin were found in a 24 year-old male patient, which were considered to be the result of a decrease in male hormones caused by pituitary dysfunction. This decrease in male hormones has been identified in a pre-employment physical examination 2 months earlier. Brain magnetic resonance imaging (MRI) was performed and hormone levels were assessed. The patient had no dizziness, headache, blurred vision, or other symptoms, and no history of hypertension, diabetes, or HIV. Physical examination on admission showed that body temperature was 36.6°C, heart rate was 74 beats/min, breathing rate was 20 beats/min, and blood pressure was 117/74 mmHg. The patient had normal development. Systemic superficial lymph nodes were negative. Binocular eye movement and light reflex were normal. Physiological reflexes were normal, and pathological reflexes were not elicited.

### 2.2. Laboratory examination

After admission, biochemical index tests, routine urine tests, and routine stool tests were conducted multiple times. The red blood cell count was 3.93 × 10^12^/L (normal range: 3–5.8 × 10^12^/L) and hemoglobin was 114 g/L (normal range: 130–175 g/L). Estradiol was 52.02 pg./mL (normal range: 0–39.8 pg./mL), prolactin was 28.47 ng/mL (normal range: 2.1–17.7 ng/mL), luteinizing hormone was <0.07 mIU/mL (normal range: 1.5–9.3 mIU/mL), follicle-stimulating hormone was <0.30 mIU/mL (normal range: 1.4–18.1 mIU/mL), progesterone was <0.21 ng/mL (normal range: 0.28–1.22 ng/mL), testosterone was 7.35 ng/dL (normal range: 123.06–813.8 ng/dL), total triiodothyronine was 0.43 ng/mL (normal range: 0.6–1.81 ng/mL), total thyroxine was 3.5 μg/dL (normal range: 3.2–12.6 μg/dL), thyroid-stimulating hormone was 2.623 mIU/L (normal range: 0.55–4.78 mIU/L), and serum cortisol (at 8:00 AM) was 12.67 μg/dL (normal range: 4.3–22.4 μg/dL). There were no signs or symptoms related to the posterior pituitary.

### 2.3. Imaging

**Figure 1:**
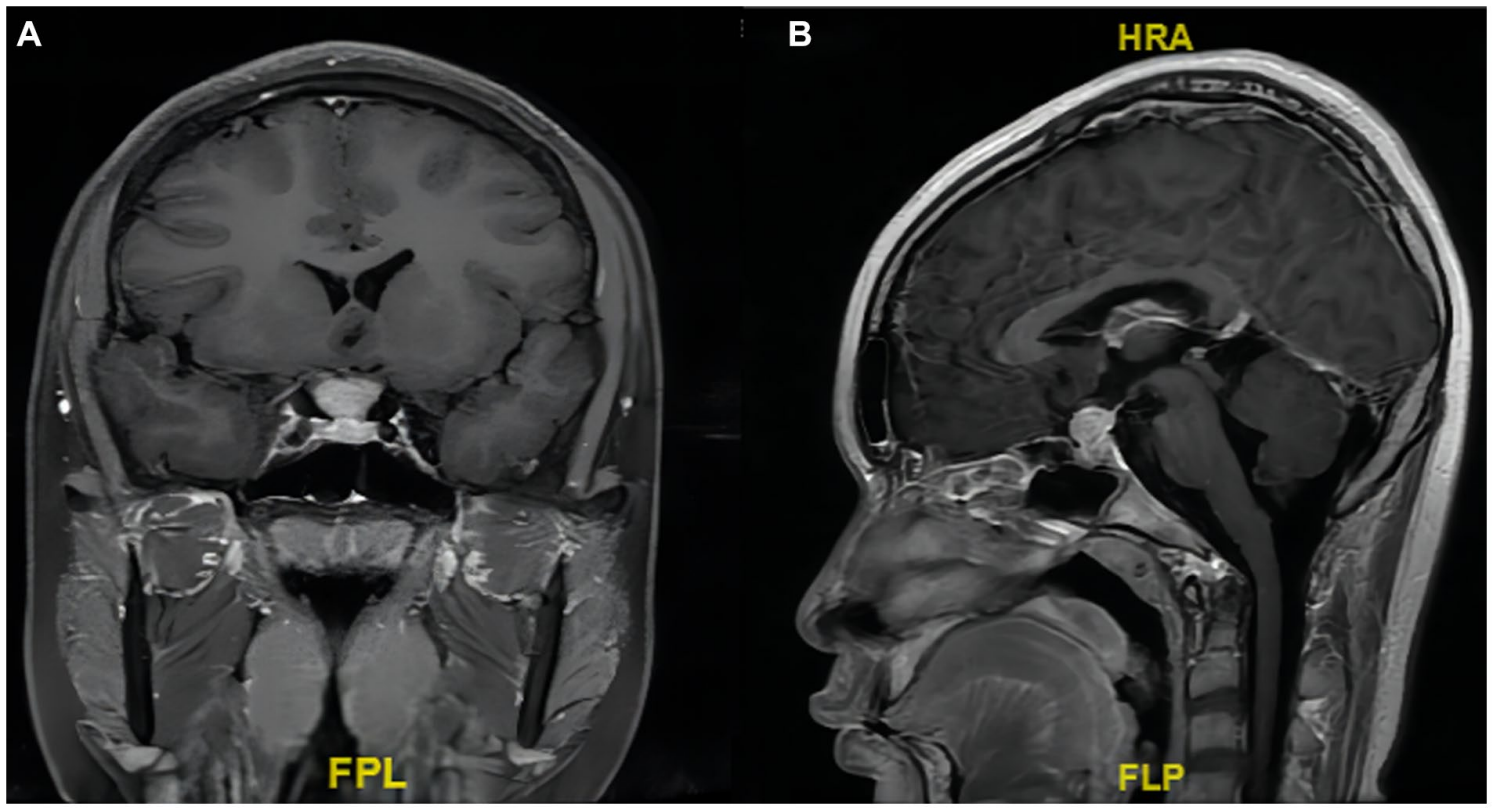
Preoperative enhanced T1-weighted MRI scan showing an enlarged pituitary stalk. **(A)** Coronal and **(B)** sagittal images showing that the tumor and the normal pituitary gland are bounded by the diaphragma sellae. The tumor size is about 12 × 15 × 16 mm.

On a contrast-enhanced MRI scan of the head, a pituitary stalk tumor exhibited isointense signals on T1- and T2-weighted images, and homogeneous contrast enhancement was observed (Figure 1). The lesion size was about 12 × 15 × 16 mm. Coronal and sagittal images showed that the tumor and the normal pituitary gland were bounded by the diaphragma sellae. The optic chiasm was slightly compressed, and the bottom of the sella sank to the left. The cavernous sinus structure on each side was clear. The eye sockets and nasopharynx were normal. The mucosae of the ethmoid sinus and left maxillary sinus were thickened. The initial diagnosis was lymphocytic hypophysitis.

### 2.4. Treatment

**Figure 2:**
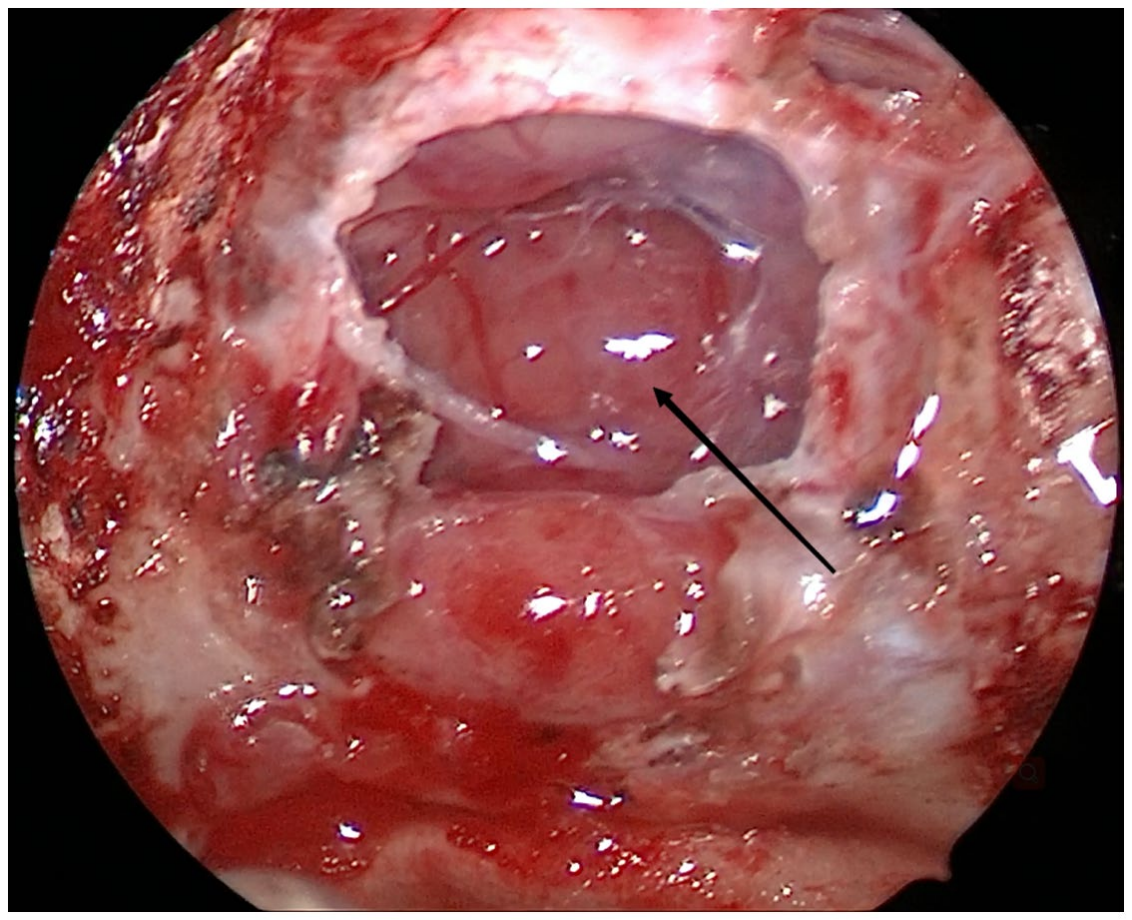
During the operation, we saw that the enlarged pituitary stalk had an abundant blood supply (arrow).

After admission for preoperative examination, the patient underwent endoscopic transnasal mass exploration. During the operation, the sellar dura was opened and normal pituitary tissue was found. The tuberculum sellae was abraded to expand the approach, and the brain was explored. This showed that the pituitary stalk had grown expansively and the blood supply was abundant (Figure 2). The tumor texture was hard. It was light pink, and it was difficult to find normal pituitary stalk tissue. The tumor was partially resected for pathological examination. The bottom of the sella was reconstructed with artificial meninges and mucosal flaps. Transient diabetes insipidus occurred after the operation, but there was no cerebrospinal fluid leakage or intracranial infection.

**Figure 3:**
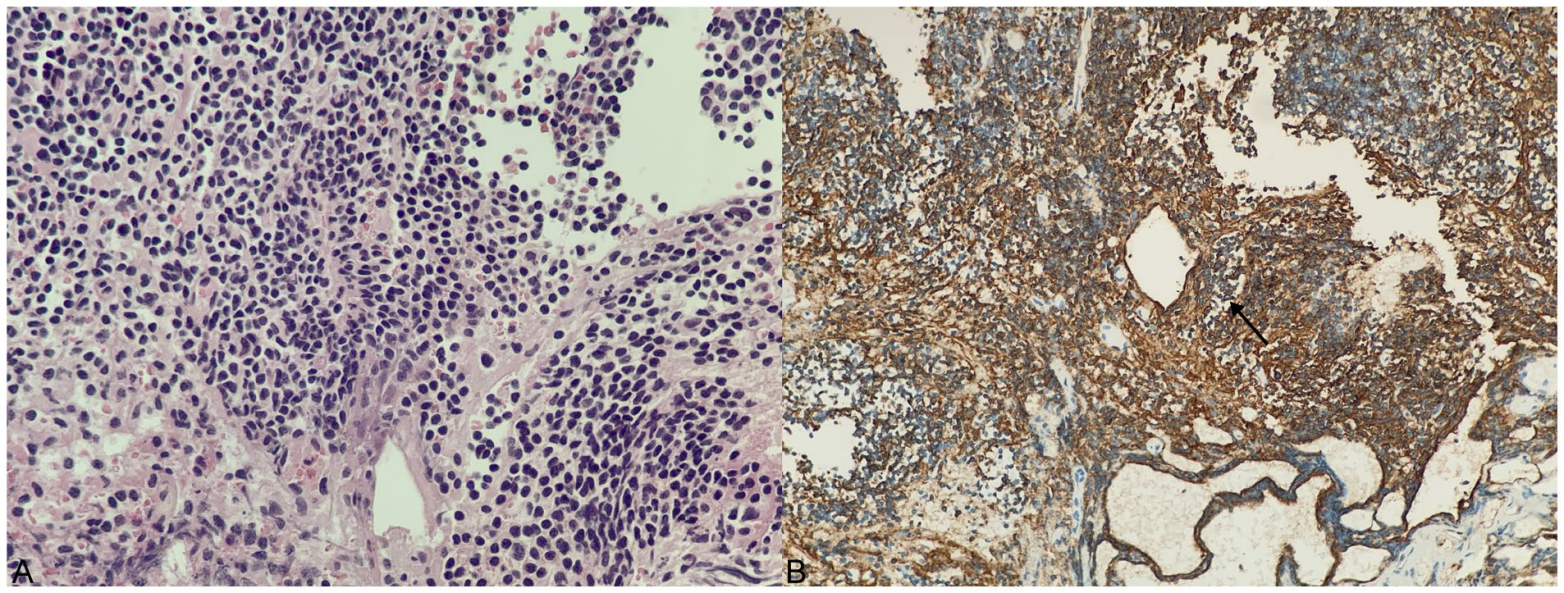
Histology and immunohistochemical staining of tumor. **(A)** H&E staining, ×400. **(B)** Substantial CD20-positive B lymphocytes (arrow shows that the cell membrane of a CD20-positive B lymphocyte is tan).

Pathological examination of the surgical specimens showed that the lymphoid tissue proliferated in the mass, and the cells in the focal area were mildly heterogeneous. Cell immunohistochemistry indicated that the surgical specimens were positive for CD20, PAX5, CD79α, CD3, CD5, CD23, CD21, Bcl-2, K, and λ. CK, CD56, Syn, CgA, SALL4, S-100, Bcl-6, CyclinD1, Langerin, CD1α, Mum-1, MPO, CD117, and EBER were negative. The Ki-67 index of cell proliferation was 40%. The pathological diagnosis was extranodal MALT of the sellar region, which is an MZL (Figure 3).

**Figure 4:**
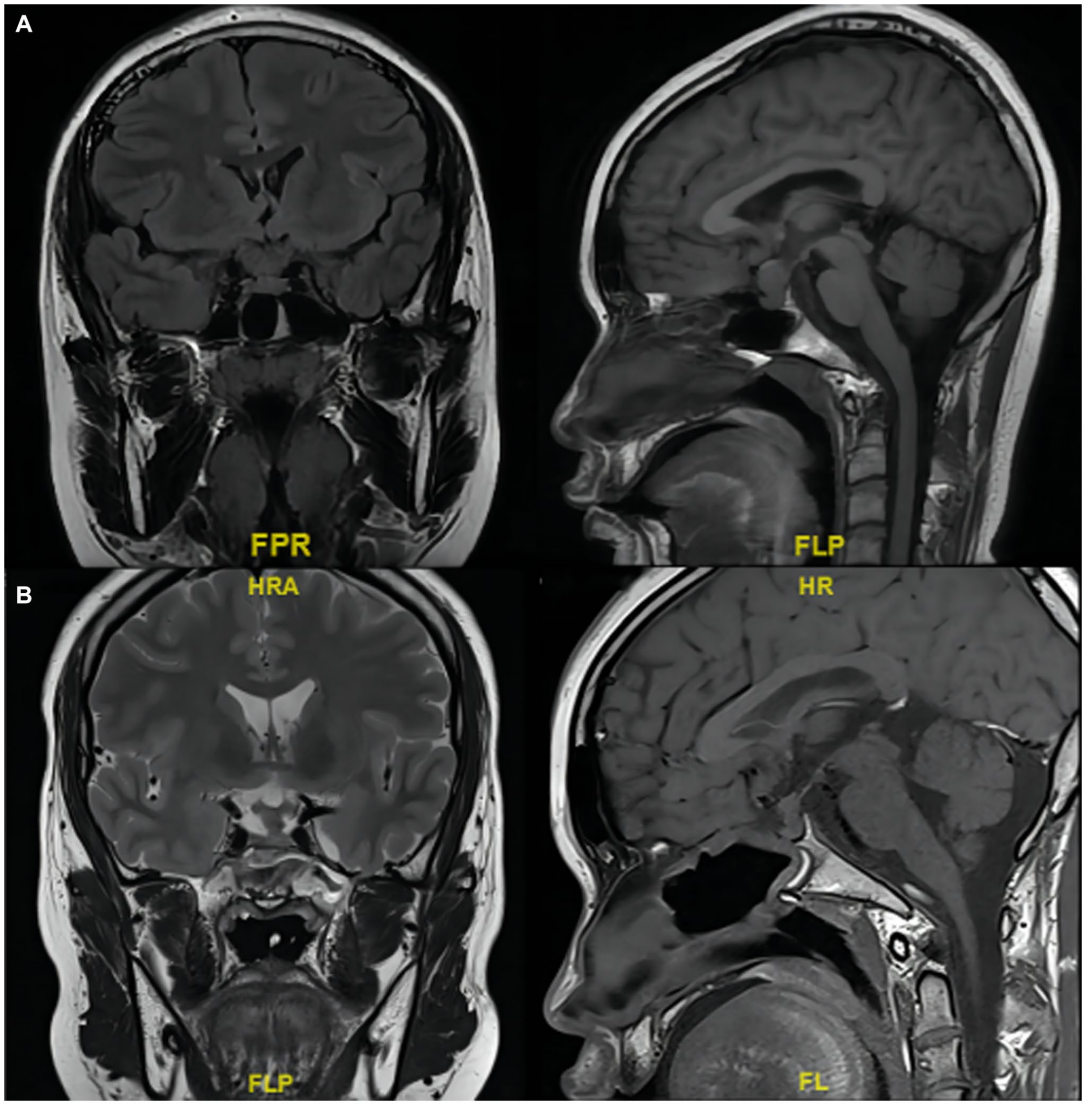
MRI scans of the tumor in the sellar region involving the pituitary stalk. **(A)** Before and **(B)** 3 months after partial resection.

The patient and family members refused radiotherapy and chemotherapy. Based on the pituitary and adrenal cortex function test results, androgen replacement therapy was given. At the follow-up 3 months later, a pituitary MRI showed that the tumor did not grow after surgery (Figure 4; the patient and his family selected only unenhanced MRI). The testosterone level was normalized (151.67 ng/dL; normal range: 123.06–813.8 ng/dL). The patient did not have symptoms such as vision defects, headache, or cranial nerve abnormalities. He was restored to a healthy life.

## 3. Discussion

Using the PubMed database, we conducted a retrospective analysis of case reports on MALT lymphoma in the sellar region from 1997 to 2022. Table 1 provides a detailed clinical summary of the ten identified patients. The median age was 50 years (range: 24–61 years). Nine were women (90%). The most common clinical symptoms were vision defects (50%) and headaches (30%). In terms of treatment, three patients (30%) received radiotherapy only, one (10%) received chemotherapy only, three (30%) received surgery and radiotherapy, two (20%) received radiotherapy and chemotherapy, and one (10%) received surgery, radiotherapy, and chemotherapy. Despite the varying treatments, the prognoses were good, indicating that MALT lymphoma is less malignant.

**Table 1:** The clinical summary of 10 immunocompetent adult sellar region MALT lymphoma patients.

Authors and year	Ref. no	Gender/age	Presenting symptoms	Site	Immunohistochemistry & molecular Studies	Treatment	Follow up
Kumar et al. (1997)	(9)	F 40	Numbness vision changes	Right cavernous sinus	Lambda light chain restriction	RT	NED at 63 mo
Sanjeevi et al. (2001)	(10)	F 46	Headache vision changes	Left cavernous sinus	L26 positive. Kappa light chain restriction. (VJ-PCR) Ig heavy chain gene rearrangement	Partial excision and RT	NED at 15 mo
Garcia-Serra et al. (2003)	(11)	F 57	Vision changes	Right cavernous sinus	Small B-Cell kappa restricted monoclonal cells that were CD10 and CD5 negative	RT (include the craniospinal axis)	NED at 24 mo
Razaq et al. (2009)	(5)	F 61	Headaches vision deficits	Cavernous sinus and optic nerve	CD20 positive. CD10 and BCL-2 negative. IgG lambda chain restricted	Rituximab and whole-brain RT	NED at 25 mo
de la Fuente et al. (2016)	(12)	F 30	Facial pain	Cavernous sinus	CD20 positive. EBER negative	Partial resection and foca RT	CR
		F 51	Focal paresthesias numbness	Left cavernous sinus	CD20 positive. EBER negative	Rituximab/bendamustine and foca RT	CR
		F 59	Headache	Suprasellar region	CD20 positive. EBER negative	Focal RT	CR
		F 48	Cranial nerve palsy	Bilateral cavernous sinus	CD20 positive. EBER negative	Rituximab	CR
		F 50	Cranial nerve palsy	Cavernous sinus	CD20 positive. EBER negative	Partial resection and Focal RT	CR
Yang et al. (2021)	(13)	M 59	Right ptosis Blurred vision	Right cavernous sinus	N/A	Subtotal resection, RT and CHT	NED at 24 mo

RT, radiation therapy; CHT, chemotherapy; NED, no evidence of disease; CR, complete response; REF, reference; MO, months; N/A, not available.

Patients with strong immunity rarely suffer from primary CNS lymphoma (PCNSL), which accounts for 4% of intracranial tumors and 4%–6% of extranodal lymphomas (14). According to epidemiological analysis, primary pituitary lymphoma (PPL) is more likely to occur in individuals aged 50–60, and the number of females is slightly higher than that of males (15). Most PPL is diffused large B-cell lymphoma (DLBCL), while MALT lymphoma is rare. MALT lymphoma is an indolent tumor that spreads slowly, and most patients have a good prognosis. It often occurs in the gastrointestinal tract, eye appendages, skin, thyroid, lungs, salivary glands, breasts, etc., while it is extremely rare in the sellar region.

The specific pathogenesis of PPL is still unclear. Some researchers believe that when normal lymphocytes enter the CNS during inflammation, the tumor is transformed, leading to PPL, or the normal lymphoid tissues in the CNS are transformed, leading to PPL (15). Inflammation and autoimmune diseases will cause chronic irritation, which may eventually lead to MALT lymphoma (16). It is worth studying whether the tumor will subside if the initial cause is eliminated.

Pathology and immunohistochemistry remain the gold standards for diagnosing PPL (5). Endoscopic transnasal transsphenoidal biopsy and stereotactic biopsy are two ways to diagnose PPL. However, it should be noted that corticosteroids, which can significantly shrink tumors, should be avoided before stereotactic biopsy. The MRI characteristic of PPL is equal or low signals on T1-and T2-weighted images (17). The T2-weighted image signal is not high as the tumor cells are dense and the nucleo-plasma ratio is high. This characteristic makes it easier to distinguish PPL from other sellar tumors (18). Nonetheless, MRI scans of patients have been reported to be varied and nonspecific. Misdiagnosis is likely to occur. The clinical symptoms of PPL are also nonspecific. Most patients with PPL often have headaches, cranial nerve abnormalities, and pituitary insufficiency (19). Headaches may occur as the sellar bone erodes, the diaphragma sellae extends, or ventriculomegaly occurs (20). Except for the sex hormone secretion disorder, no other symptoms or signs were found in the patient. In summary, it is difficult to diagnose PPL clinically based on symptoms and signs (including MRI scans).

PPL should be managed based on the protocol for treating PCNSL, including surgery, chemotherapy, and/or radiotherapy (15, 21). As PPL is invasive and multifocal, the lesion cannot be completely removed by surgery. The main purpose of the surgery is to relieve local compression and confirm the diagnosis by biopsy. However, Weller et al. confirmed that the progression-free and overall survival of patients undergoing complete or subtotal resection are higher than those undergoing biopsy (22). Comprehensive treatment with chemotherapy as the main treatment and radiotherapy as the auxiliary treatment is effective for PCNSL. High-dose methotrexate-based systemic chemotherapy improves the survival rate of PCNSL patients (23–26). Additionally, combination treatment involving rituximab/temozolomide±interferon-beta is also effective for patients with relapsing and refractory PCNSL (23).

In the current case, the tumor in the sellar region originated from the pituitary stalk. Blindly removing the tumor would cut off the connection between the pituitary and hypothalamus, which would eventually lead to a hormone disorder that would necessitate lifelong hormone replacement therapy. Therefore, we performed partial resection and then performed pathological examination to confirm the diagnosis. After the diagnosis, the patient and family members refused radiotherapy and chemotherapy. They only agreed to androgen therapy for the sex hormone disorder.

## 4. Conclusion

Primary sellar region MALT lymphoma is a rare illness. Identification of more cases is required to study its pathogenesis and formulate better treatment plans. In the current case, the patient did not undergo radiotherapy or chemotherapy after partial resection. Close follow-up is needed, and the next treatment plan will be formulated based on the patient’s condition. In summary, MRI scans and clinical symptoms of sellar region MALT lymphoma lack specificity. Neurosurgeons should pay attention to this rare disease in the differential diagnosis of sellar disease, as timely diagnosis and treatment can significantly improve the patient’s prognosis.

## Data availability statement

The original contributions presented in the study are included in the article/Supplementary material, further inquiries can be directed to the corresponding author.

## Ethics statement

Written informed consent was obtained from the individual(s) for the publication of any potentially identifiable images or data included in this article.

## Author contributions

SC was responsible for methodology, data curation, resources, formal analysis, and writing – original draft. JX, PC, and HL were responsible for supervision and writing – review and editing. ZC was responsible for ensuring that the descriptions are accurate and agreed on by all authors. All authors contributed to the article and approved the submitted version.

## Funding

This work was supported by the Major Research Projects of the Chinese Academy of Traditional Chinese Medicine (grant no. ZZ15-WT-04); the Science and Technology Research Project of Jiangxi Provincial Department of Education (grant no. GJJ200133); and the Science and Technology Plan of Jiangxi Provincial Health Commission (grant no. 202130447).

## Conflict of interest

The authors declare that the research was conducted in the absence of any commercial or financial relationships that could be construed as a potential conflict of interest.

## Publisher’s note

All claims expressed in this article are solely those of the authors and do not necessarily represent those of their affiliated organizations, or those of the publisher, the editors and the reviewers. Any product that may be evaluated in this article, or claim that may be made by its manufacturer, is not guaranteed or endorsed by the publisher.
