# Supplementary material for: Primary pituitary stalk mucosa-associated lymphoid tissue lymphoma: a case report and literature review
Source: Front Neurol. 2023 Jul 6;14:1193391. doi: 10.3389/fneur.2023.1193391 (PMC10406508; doi:10.3389/fneur.2023.1193391)
Supplement: Supplementary file 1 [file Table_1.DOCX]

***TABLE 1*** *The clinical summary of 10 immunocompetent adult sellar region MALT lymphoma patients.*

| **Authors and year** | **Ref. no** | **Gender/ age** | **Presenting symptoms** | **Site** | **Immunohistochemistry**  **&Molecular Studes** | **Treatment** | **Follow up** |
| --- | --- | --- | --- | --- | --- | --- | --- |
| Kumar et al.,1997 | 9 | F 40 | Numbness  Vision changes | Right cavernous sinus | Lambda light chain restriction | RT | NED at 63 mo |
| Sanjeevi et al.,2001 | 10 | F 46 | Headache  Vision changes | Left cavernous sinus | L26 positive. Kappa light chain restriction. (VJ-PCR) Ig heavy chain gene rearrangement | Partial excision and RT | NED at 15 mo |
| Garcia-Serra et al.,2003 | 11 | F 57 | Vision changes | Right cavernous sinus | Small B-Cell kappa restricted monoclonal cells that were CD10 and CD5 negative | RT(include the craniospinal axis) | NED at 24 mo |
| Razaq et al.,2009 | 5 | F 61 | Headaches  Vision deficits | Cavernous sinus and optic nerve | CD20 positive. CD10 and BCL-2 negative. IgG Lambda chain restricted | Rituximab  and whole-brain RT | NED at 25 mo |
| de la Fuente et al.,2016 | 12 | F 30 | Facial pain | Cavernous sinus | CD20 positive. EBER negative | Partial resection  and Foca RT | CR |
|  |  | F 51 | Focal paresthesias Numbness | Left cavernous sinus | CD20 positive. EBER negative | Rituximab/bendamustine and Foca RT | CR |
|  |  | F 59 | Headache | Suprasellar region | CD20 positive. EBER negative | Focal RT | CR |
|  |  | F 48 | Cranial nerve palsy | Bilateral cavernous sinus | CD20 positive. EBER negative | Rituximab | CR |
|  |  | F 50 | Cranial nerve palsy | Cavernous sinus | CD20 positive. EBER negative | Partial resection  and Focal RT | CR |
| Yang et al.,2021 | 13 | M 59 | Right ptosis  Blurred vision | Right cavernous sinus | N/A | Subtotal resection、RT  and CHT | NED at 24 mo |

*RT=radiation therapy; CHT= chemotherapy; NED=no evidence of disease; CR=complete response; REF=reference; MO=months; N/A=not available.*
